# Long term effects of digital education among healthcare professionals in paediatric dermatology: Opportunities for improving care

**DOI:** 10.1002/ski2.143

**Published:** 2022-07-20

**Authors:** Aviël Ragamin, Renske Schappin, Willemijn C. A. M. Witkam, Magda Spiering, Elodie Mendels, Marie L. A. Schuttelaar, Suzanne G. M. A. Pasmans

**Affiliations:** ^1^ Department Dermatology, Center of Pediatric Dermatology Erasmus MC University Medical Center‐Sophia Children's Hospital Rotterdam The Netherlands; ^2^ Department of Dermatology Erasmus MC University Medical Center Rotterdam Rotterdam The Netherlands; ^3^ Department of Dermatology University Medical Center Groningen, University of Groningen Groningen The Netherlands

## Abstract

**Background:**

Topical corticosteroids (TCS) are the cornerstone of treatment for patients with atopic dermatitis (AD). Unfortunately, anxiety and misplaced beliefs on TCS, known as corticophobia, is common among health care professionals (HCPs) and could influence their practices, resulting in suboptimal patient care.

**Objectives:**

To investigate the effects of digital education (DE) on the knowledge of TCS, practices and corticophobia among HCPs in paediatric dermatology.

**Methods:**

HCPs registered for an interactive online masterclass on paediatric dermatology including the treatment of AD and TCS were invited to participate in a survey on knowledge of TCS, self‐reported practices and corticophobia. Corticophobia was measured using the TOPICOP‐P questionnaire (range: 0%–100%, with higher scores indicating more corticophobia). Participants received the survey before, directly after, and 6 months after DE.

**Results:**

Of the 86 participants, 66 (77%) completed the survey before the masterclass, 76 (88%) directly after, and 34 (40%) 6 months after. Key components of knowledge on TCS and self‐reported practices improved greatly after DE, such as correct prescription amount of TCS (45%, 91%, 88%) and application instructions (56%, 99%, 94%). Overall corticophobia decreased after DE with median scores dropping from 33% before DE to 25% after DE (*p* < 0.01) and remained 25% 6 months later.

**Conclusion:**

Interactive DE for HCPs is an efficient tool to attain prolonged improvements of knowledge on TCS, practices, and corticophobia. All these factors are important for optimal care for patients. This study shows great opportunities for improving care by investing in HCPs.

1

**What is already known about this topic?**

- Topical corticosteroids are the cornerstone of treatment for patients with atopic dermatitis. Unfortunately, anxiety and misplaced beliefs on topical corticosteroids, also known as corticophobia, is common among health care professionals and could influence their practices, resulting in suboptimal care.
- Digital education is increasingly offered to health care professionals in general. The short term effects of digital education have been demonstrated before.

**What does this study add?**

- This study demonstrates that interactive digital education for healthcare professionals is an efficient tool to attain prolonged improvements in knowledge on topical corticosteroids, practices and corticophobia.
- Additionally, this study shows great opportunities for improving care by investing in and empowering healthcare professionals.

## 2 INTRODUCTION

Atopic dermatitis (AD), also known as atopic eczema, is a common chronic inflammatory disease of the skin.^1^ It is characterized by chronic inflammatory skin resulting in intense itch and red skin lesions. With a prevalence up to 20%, AD is common in children.^2^

Topical corticosteroids (TCSS) are the cornerstone of treatment for children with AD.^3^, ^4^ Although safety and efficacy of TCS are well‐studied, HCPs can experience fear and anxiety for potential side effects caused by TCSS, also known as corticophobia.^5^, ^6^, ^7^, ^8^, ^9^ Anxiety and misplaced beliefs among HCPs could influence prescription practices and patient education.^10^ Reluctance to treat AD with TCS could lead to greater disease burden, whereas unintentional risk messages about TCS during patient education could reduce treatment adherence.^11^, ^12^ To improve adherence and disease control in AD, uniformity in prescription practices and education about TCS must be achieved among HCPs that interact with patients with AD.

In the Netherlands, most children with AD are treated with TCS by general practitioners and pediatricians.^13^ Additionally, youth health care physicians have an important role in primary detection of AD and education of caregivers to use emollients and to adhere to the TC, as they perform regular developmental check‐ups,^14^ see Appendix S1. Lastly, (district) nurses and nurse practitioners play an essential role in paediatric dermatology as they educate patients and in the case of nurse practitioners, prescribe treatment.^15^, ^16^ Unfortunately, these groups receive limited dermatological education during training.

The short‐term effects of additional education on TCS for pharmacists have been demonstrated before.^17^ However, the effects of digital educational interventions aimed at improving knowledge, practices and corticophobia among HCPs that prescribe, treat and educate patients with AD is still lacking. For other diseases, digital education seems to be a (cost)‐effective tool to improve practices on the short term.^18^, ^19^ However, improving practices in AD may be a greater challenge as non‐dermatologists have more anxiety for TCS.^9^ Digital education, should therefore not only improve knowledge, but the anxiety as well. Furthermore, the long‐term effects of digital education are still unclear.

In this study, we therefore aim to investigate the effects of interactive digital education for professionals in paediatric dermatology by assessing key components of knowledge on TCS, practices, and corticophobia with regards to treatment of children with AD.

## 3 MATERIALS AND METHODS

### 3.1 Setting

Health care professionals registered for an interactive online masterclass on paediatric dermatology (Dutch: Kinderdermatologie: Masterclass voor zorgprofessionals in de Jeugdgezondheidzorg – SCEM – 2020^20^) were asked to participate anonymously in a survey on atopic dermatitis in children in October 2020 in the Netherlands. During this masterclass, interactive digital education was provided by a team of paediatric dermatologists, a paediatric psychologist, and a nurse practitioner based on the National AD guidelines.^21^, ^22^ One plenary session of 45 min specifically addressed treatment of AD. During this session participants received information on the aetiology of AD, emollients and TCS (including key aspects such as the fingertip unit (FTU), a rule for the amount of TCS that needs to be applied, tapering schedules, potency classes, and side effects of TCS). Participants were asked to complete a survey before, immediately after, and 6 months after the masterclass. Invitations for the survey were sent by email, the survey was designed in Google Forms.

### 3.2 Survey

The anonymous survey contained several topics, including socio‐demographics, knowledge, self‐reported practices and corticophobia. After 6 months, the survey contained additional questions to evaluate the effects of digital education, asking participants whether their beliefs and practices changed. Questions were asked in a specific order to reduce order effects.

Knowledge was assessed with questions covering important aspects of topical treatment such as the FTU, different potency classes of TCS, and tapering schedules.^3^, ^23^, ^24^ Practices were assessed with questions covering important application instructions and amount of TCS needed for treatment. Beliefs on TCS and corticophobia were investigated with the Topical Corticosteroid Phobia questionnaire for professionals (TOPICOP‐P).^9^, ^25^ This questionnaire was originally developed for patients with AD, but has previously been modified for healthcare professionals.^9^ The questionnaire consists of 12 items with a 4‐point scale that address two important dimensions “worries” (6 items) and “beliefs” (6 items) on TCS.^9^, ^25^ Although, other studies report an additional subscale (fear) of the TOPICOP questionnaire, we followed the original and statistically substantiated calculation that divides the TOPICOP questionnaire into two subscales. A score ranging from 0% to 100% can be calculated for each dimension and for the total scale, with higher scores reflecting more severe corticophobia. An overview of the complete survey is provided in the Appendix S2.

### 3.3 Statistical analysis

Responses were analyzed using SPSS software (version 26; IBM Corp). Differences between pre‐education, post‐education and 6 months thereafter were analyzed with independent *t*‐tests for normally distributed interval variables, Mann‐Whitney U tests for not normally distributed variables, chi‐square test for categorical variables with >2 variables and Fisher's exact for dichotomous variables. Additionally, Spearman's coefficients (for Mann–Whitney *U* tests) and Cramer's V (for chi‐square and Fisher's exact) were calculated to determine effect sizes. An effect size of 0.10 was considered a small effect, 0.30 a medium effect, and 0.50 a large effect, for Cramer's V effect size interpretation was adjusted when degrees of freedom >1.^26^ For subgroup analyses, participants were divided in three subgroups: Pediatricians (consisting of pediatricians and residents in paediatrics), youth health care physicians (consisting of physicians and residents in youth health care), and nurses (consisting of nurses and nurse practitioners). A two‐sided *p* < 0.05 was considered statistically significant.

## 4 RESULTS

### 4.1 Demographic characteristics

A total of 86 HCPs participated in the online masterclass. The questionnaire was completed 176 times: 66 times before, 76 times directly after, and 34 times 6 months after the online masterclass (Table 1). This resulted in a response rate of 77% before, 88% directly after, and 40% 6 months after the masterclass. The majority of the responders were females and the median age was approximately 45 years. Youth health care physicians formed the largest group of responders. Almost half of the responders prescribed TCS or gave instructions on TCS use on a daily or weekly basis. No significant differences in age, gender, profession, and prescription frequency were found between responders before and after the online masterclass. Medical guidelines were the most common source of information on TCS. The proportion of responders that reported masterclasses and conferences as an important source of information increased after the masterclass from 57% to 81% directly after the masterclass and increased further to 100% (*p* < 0.01) 6 months after the online masterclass.

**TABLE 1:** Overview of participant characteristics

Item	Pre‐education (*n* = 66)	Post‐education (*n* = 76)	Follow‐up after 6 months (*n* = 34)	Sig. (*p* value)		
				Pre versus Post^a^	Pre versus Follow‐up^b^	Post versus Follow‐up^c^
Age, year (95% CI)	45 (43–48)	45 (43–47)	45 (42–48)	0.98	0.98	0.89
Female, no (%)	57 (89)	70 (92)	32 (97)	0.54	0.26	0.67
Profession, no (%)				0.92	0.47	0.29
Pediatricians	19 (30)	25 (33)	6 (18)			
Youth health care physicians	31 (48)	35 (46)	19 (56)			
Nurses^d^	14 (22)	16 (21)	8 (24)			
TCS prescription or education frequency, no (%)				0.90	0.60	0.63
(Almost) never	18 (29)	21 (28)	11 (35)			
Monthly	16 (25)	23 (30)	7 (23)			
Weekly	23 (37)	24 (32)	8 (26)			
Daily	6 (10)	8 (11)	5 (16)			
Source of information on TCS, no (%)						
Guidelines	42 (70)	60 (81)	26 (79)	0.16	0.47	0.80
Evidence based journals	14 (23)	22 (29)	7 (21)	0.44	1.0	0.48
Own experience	31 (52)	37 (50)	10 (30)	0.86	0.05	0.09
Masterclasses, congresses, etc.	34 (57)	60 (81)	33 (100)	**<0.01**	**<0.01**	**<0.01**
Information pamphlets	9 (15)	14 (9)	2 (6)	0.65	0.32	0.14
Commercial material	0 (0)	1 (1)	1 (3)	1.0	0.36	0.52

*Note*: Percentages and numbers may not add up because of missing values.

Abbreviation: TCS, topical corticosteroids. Bold values indicate statistically significant outcomes (*p* ≤0.05)

^a^ Responses before digital education compared to responses directly after digital education.

^b^ Responses before digital education compared to responses 6 months after education.

^c^ Responses directly after digital education compared to responses 6 months after education.

^d^ Nurses consisted out of nurses and nurse practitioners (2 pre and post‐education, 1 after 6 months of follow‐up). An effect size of 0.10 is considered small, 0.30 medium, and 0.50 large.^26^

### 4.2 Effects of digital education on knowledge and self‐reported practices

Familiarity with all key aspects of TCS, such as the FTU, TCS potency classes, and tapering schemes all increased significantly after digital education (Table 2). These effects remained after 6 months. In addition, improvement of patient education concerning the application instructions and amount TCS improved had a large effect size directly after digital education. Professionals kept giving correct instructions after 6 months. A ceiling effect was detected for treatment duration, with already almost 100% of professionals providing correct information before digital education.

**TABLE 2:** Effects of digital education on prescription practices and patient education

Item	Pre‐education	Post‐education	Six months after education	Pre versus Post^a^		Pre versus Follow‐up^b^		Post versus Follow‐up^c^	
	*N* (%)	*N* (%)	*N* (%)	Effect size (*φ*c)	*p*	Effect size (*φ*c)	*p*	Effect size (*φ*c)	*p*
Familiarity with FTU	43 (69)	76 (100)	33 (100)	**0.44**	**<0.01**	**0.37**	**<0.01**	‐	‐
Familiarity with classes of potency	48 (76)	75 (99)	31 (94)	**0.35**	**<0.01**	0.22	0.05	0.13	0.22
Familiarity with tapering schemes	56 (88)	75 (99)	32 (97)	**0.23**	**0.01**	0.16	0.16	0.06	0.50
Duration of a tube TCS				**0.50**	**<0.01**	**0.42**	**<0.01**	0.04	0.73
1–2 weeks^d^	27 (45)	69 (91)	29 (88)						
1 month or longer	33 (55)	7 (9)	4 (12)						
Treatment duration of TCS				**0.21**	**0.03**	0.16	0.29	‐	‐
Until patches are gone^d^	50 (93)	73 (100)	33 (100)						
Maximum duration 2–4 weeks	2 (7)	0 (0)	0 (0)						
Application instructions				**0.53**	**<0.01**	**0.40**	**<0.01**	0.13	0.22
No instructions	15 (24)	1 (1)	2 (7)						
Thin	13 (21)	0 (0)	0 (0)						
According FTU^d^	35 (56)	75 (99)	31 (94)						
Thick	0 (0)	0 (0)	0 (0)						

*Note*: Percentages and numbers may not add up because of missing values.

Abbreviation: TCS, topical corticosteroids. Bold values indicate statistically significant outcomes (*p* ≤0.05)

^a^ Responses before digital education compared to responses directly after digital education.

^b^ Responses before digital education compared to responses 6 months after education.

^c^ Responses directly after digital education compared to responses 6 months after education. An effect size of 0.10 is considered small, 0.30 medium, and 0.50 large.^26^

^d^ Correct response.

### 4.3 Effects on corticophobia

Overall corticophobia significantly decreased after digital education and this effect remained stable over the course of 6 months, Table 3. Further analysis shows a significant improvement in overall beliefs of physicians in public health and in nurses. No significant effects were found on the worries subscale among all professionals. However, the medium effect size suggests that these findings may not be significant due to a relative small sample size.

**TABLE 3:** Effects of digital education on corticophobia

	Pre‐education		Post‐education		Six months after education		Pre versus Post^a^		Pre versus Follow‐up^b^		Post versus Follow‐up^c^	
Corticophobia	*N*	Median % (IQR)	*N*	Median % (IQR)	*N*	Median % (IQR)	Effect size (r)	*p*	Effect size (r)	*p*	Effect size (r)	*p*
Total corticophobia												
Overall	57	33 (28–36)	70	25 (19–33)	32	25 (17–33)	**0.34**	**<0.01**	**0.31**	**<0.01**	0.01	0.91
Pediatricians	18	33 (25–38)	24	24 (17–31)	6	17 (14–28)	0.38	0.09	**0.55**	**0.01**	0.30	0.67
YH Physicians	26	31 (26–36)	32	25 (17–33)	18	25 (17–33)	**0.22**	**0.02**	0.15	0.33	0.06	0.12
Nurses	13	39 (31–46)	14	24 (19–30)	8	28 (19–35)	**0.50**	**0.03**	0.36	0.10	0.19	0.40
‘Beliefs’												
Overall	63	33 (22–39)	76	28 (11–33)	32	28 (11–33)	**0.24**	**<0.01**	**0.27**	**<0.01**	0.03	0.78
Pediatrics	18	33 (22–39)	24	28 (11–33)	6	11 (4–33)	0.31	0.35	0.49	0.14	0.20	0.86
YH Physicians	26	28 (17–39)	32	28 (11–39)	18	31 (11–33)	0.12	0.04	0.06	0.65	0.02	0.29
Nurses^d^	13	42 (33–50)	14	28 (11–39)	8	33 (15–33)	0.45	**0.05**	**0.56**	**0.01**	0.01	0.97
‘Worries’												
Overall	58	33 (22–44)	70	28 (17–33)	33	28 (22–33)	**0.26**	**<0.01**	**0.21**	**0.05**	0.05	0.58
Pediatricians	18	31 (22–46)	24	28 (18–33)	6	22 (17–29)	0.28	0.09	0.37	0.08	0.17	0.61
YH Physicians	27	33 (22–39)	32	28 (22–33)	18	28 (22–35)	0.22	0.07	0.16	0.27	0.07	0.40
Nurses^d^	13	33 (22–50)	14	22 (15–33)	8	25 (22–33)	0.35	0.09	0.13	0.60	0.23	0.30

*Note*: Percentages may not add up because of missing values.

Abbreviation: YH, Youth health care. Bold values indicate statistically significant outcomes (*p* ≤0.05)

^a^ Responses before digital education compared to responses directly after digital education.

^b^ Responses before digital education compared to responses 6 months after education.

^c^ Responses directly after digital education compared to responses 6 months after education. The Mann–Whitney *U* test was used for all the analyses.

^d^Nurses consisted out of nurses and nurse practitioners (2 pre and post‐education, 1 after 6 months of follow‐up). TOPICOP‐P scores and subscores can range from 0 to 100. Higher scores imply more corticophobia. An effect size of 0.10 is considered small, 0.30 medium, and 0.50 large.^26^

### 4.4 Effects of education after 6 months

After 6 months, the majority of responders (68%) reported that their practice patterns or patient education changed or felt empowered after digital education, see Appendix (Table S4). An increase in patient education on the use of TCS was reported by 39% of HCPs. Only a small portion (10%) of participating HCPs reported to refer less patients. Although, the response rate for the other statements was significantly lower (only 28%), the answers provide some relevant insights in the perceived effects of digital education. HCPs reported to make more use of tapering schedules (44%), prescribed more potent (22%) and more TCS (22%). Finally, almost all responders (89%) felt more competent with TCS treatment of AD.

## 5 DISCUSSION

In this study, we investigated the effects of interactive digital education on knowledge of TCS, self‐reported practices, and corticophobia among HCPs involved in care for children with AD. After education, HCPs showed prolonged improvements in knowledge on TCS and practices, and less corticophobia. Participating HCPs underlined the added value of the interactive digital education. Our data suggest that interactive digital education for HCPs is an efficient tool to attain prolonged improvements among HCPs.

### 5.1 Effects of digital education

Digital education is an effective tool to improve knowledge on TCS, practices, and corticophobia among HCPs involved in care for children with AD and in general.

First of all, we found significant and large improvements in knowledge on TCS and self‐reported practices. These findings are in line with previous research investigating prescription behaviour of antibiotics for respiratory infections in primary care.^19^, ^27^ Additionally, digital education improved beliefs of HCPs. Prior education, we found similar beliefs and anxiety for TCS, expressed as corticophobia scores, as compared to previous research among nurses and pediatricians.^8^, ^9^ After education, we found approximately 20% decrease in corticophobia, leading beliefs to be comparable to dermatologists, which can be seen as the gold standard.^9^ The size of this improvement is similar to a personalized face‐to‐face intervention targeted at pharmacists, adding support to the case for digital education as an efficient tool.^28^ Furthermore, almost all responders felt more competent with treatment of AD after digital interactive education. Finally, we report lasting effects of digital education on knowledge of TCS, practices, and corticophobia of HCPs. Although, other research on the long‐term effects of digital education is lacking, our results suggest prolonged effects of digital education. More research will be needed to help fill the gap concerning the long‐term effects of digital education, preferably based on patient outcomes in clinical trials. For now, digital interactive education seems to be an efficient tool to improve knowledge, practices and corticophobia among HCPs involved in care for atopic dermatitis and in general.

### 5.2 Opportunity to improve care for atopic dermatitis

Guideline adherence among HCPs in paediatric dermatology should be improved to improve care for children with AD. In our study, we investigated adherence to key guideline recommendations for AD. Prior to digital education, only half of HCPs used TCS as recommended by clinical guidelines. Additionally, a large proportion of HCPs were unfamiliar with key aspects of treatment such as the existence of different TC potency classes. Other studies investigating guideline adherence among HCPs in paediatric dermatology are lacking. However, studies investigating anxiety with regards to TCS show great differences between dermatologist and non‐dermatologist, suggesting similar findings.^9^ Keeping in mind that most HCPs treat AD or provide instructions on a weekly basis, improving care at this level may be a necessity to improve care. Several barriers may prevent HCPs from adhering to guidelines.^29^ In particular anxiety and negative beliefs on TCS, corticophobia, may contribute to less guideline adherence.^9^ However, more research is needed to identify barriers to guideline adherence and treatment differences across HCPs involved in order to improve care for AD.

Fortunately, after digital education almost all HCPs adopted guideline recommendations and familiarity with all key aspects increased. This suggests that adherence to AD guidelines can be improved. Ultimately, improving care at this level is needed for more adequately treatment of AD and improve treatment adherence among children and their caretakers. Investing in HCPs could lower health care consumption and cost as less referrals and follow‐up visits may be needed. Improving guideline adherence among HCPs may be one of the most efficient tools to improve care.

### 5.3 Limitations

Some limitations of this study need to be addressed. First, this is a questionnaire‐based study and hence, real‐life practices were not assessed. Differences in self‐reported practices and real‐life practices may exist. Professional desirability bias may have occurred. Second, selection bias may have occurred as HCPs with a specific interest in paediatric dermatology may have participated in the master class. Subsequently, practices among professionals in general may even deviate further from clinical guidelines and corticophobia scores may be underestimated in this study. Thirdly, due to a relative low response rate 6 months after the master class, we cannot exclude if selection bias may have occurred and if only HCPs already familiar with all guideline recommendations participated. However, the majority of participants reported a change in practices or felt empowered after 6 months, suggesting that participants were previously unfamiliar guideline recommendations. Finally, a relative small sample size was included and this study lacked participation of general practitioners. Due to the interactive nature and specific aim of the digital master class a larger sample was not feasible.

### 5.4 Implications

Our study provides novel insights in the effects of interactive digital education for HCPs. In particular, this study shows that long lasting changes in knowledge of TCS, practices and corticophobia can be achieved, expecting to result in better care. HCPs themselves have a strong preference for interactive educational meetings to improve guideline adherence.^30^, ^31^ Policy makers, medical associations, and specialists may use these findings to develop strategies for better implementation of evidence based clinical guidelines.

In addition, our results emphasize the opportunity of improving guideline adaptation. HCPs and policy makers should realize that the vast majority of patients with (mild) AD are treated by general practitioners, pediatricians, nurses, or physicians in primary health care. In these settings, TCS are most important part of treatment.^3^ For patients with moderate to severe AD also new therapies (i.e. biologicals or JAK inhibitors) will benefit individual patients. However, at a national level, patients with AD may benefit more from improving knowledge on existing treatments with TCS.^32^ Non‐dermatologists provide a crucial role in patient education and can have a major impact on attitude towards TCS.^7^, ^33^ In addition, education may lower healthcare costs, as fewer referrals may be needed. The results of this study imply a call to invest in additional training of HCPs in (paediatric) dermatology.

## 6 CONCLUSION

This study demonstrates the long‐term positive effects of digital education for HCPs. After digital education almost all HCPs reported to follow key recommendations of clinical guidelines. In addition, beliefs on TCS improved and corticophobia reduced to levels similar to dermatologists. These effects remained stable over the course of 6 months. This study emphasizes the necessity and opportunity to improve care for patients with AD by investing in interactive digital education to empower HCPs.

## AUTHOR CONTRIBUTIONS

**Aviel Ragamin**: Conceptualization (equal); Data curation (lead); Formal analysis (equal); Methodology (equal); Writing – original draft (lead); Writing – review & editing (lead). **Renske Schappin**: Formal analysis (equal); Supervision (equal); Writing – original draft (supporting); Writing – review & editing (supporting). **Willemijn C. A. M. Witkam**: Formal analysis (supporting); Writing – original draft (supporting); Writing – review & editing (supporting). **Magda Spierings**: Writing – original draft (supporting); Writing – review & editing (supporting). **Elodie Mendels**: Writing – original draft (supporting); Writing – review & editing (supporting). **Marie L. A. Schuttelaar**: Supervision (supporting); Writing – original draft (supporting); Writing – review & editing (supporting). **Suzanne G. M. A. Pasmans**: Conceptualization (equal); Methodology (equal); Supervision (equal); Writing – review & editing (supporting).

## CONFLICT OF INTEREST

The author declares that there is no conflict of interest that could be perceived as prejudicing the impartiality of the research reported.

## ETHICS STATEMENT

This study was exempt from the Dutch Medical Research Involving Human Subjects Act according to the institutional review board of Erasmus MC (MEC‐2020‐0697).

## Supporting information

Supporting Information S1

## Data Availability

The data that support the findings of this study are not publicly available but are available from Suzanne G. M. A. Pasmans (s.pasmans@erasmusmc.nl) upon reasonable request.

## References

[1] Bieber T . Atopic dermatitis. N Engl J Med. 2008;358(14):1483–94. 10.1056/nejmra074081 18385500

[2] Bylund S , von Kobyletzki LB , Svalstedt M , Svensson Å . Prevalence and incidence of atopic dermatitis: a systematic review. Acta Derm Venereol. 2020;100(12):adv00160. 10.2340/00015555-3510 32412646 PMC9189744

[3] Eichenfield LF , Tom WL , Berger TG , Krol A , Paller AS , Schwarzenberger K , et al. Guidelines of care for the management of atopic dermatitis: section 2. Management and treatment of atopic dermatitis with topical therapies. J Am Acad Dermatol. 2014;71(1):116–32. 10.1016/j.jaad.2014.03.023 24813302 PMC4326095

[4] Leidraad NVDV . Dermatocorticosteroïden. Utrecht: Dutch Society for Dermatology and Venereology (NVDV); 2019.

[5] Siegfried EC , Jaworski JC , Kaiser JD , Hebert AA . Systematic review of published trials: long‐term safety of topical corticosteroids and topical calcineurin inhibitors in pediatric patients with atopic dermatitis. BMC Pediatr. 2016;16(1):75. 10.1186/s12887-016-0607-9 27267134 PMC4895880

[6] El Hachem M , Gesualdo F , Ricci G , Diociaiuti A , Giraldi L , Ametrano O , et al. Topical corticosteroid phobia in parents of pediatric patients with atopic dermatitis: a multicentre survey. Ital J Pediatr. 2017;43(1):22. 10.1186/s13052-017-0330-7 28245844 PMC5330138

[7] Smith SD , Hong E , Fearns S , Blaszczynski A , Fischer G . Corticosteroid phobia and other confounders in the treatment of childhood atopic dermatitis explored using parent focus groups. Australas J Dermatol. 2010;51(3):168–74. 10.1111/j.1440-0960.2010.00636.x 20695854

[8] Bos B , Antonescu I , Osinga H , Veenje S , de Jong K , de Vries TW , et al. Corticosteroid phobia (corticophobia) in parents of young children with atopic dermatitis and their health care providers. Pediatr Dermatol. 2019;36(1):100–4. 10.1111/pde.13698 30338542

[9] Lambrechts L , Gilissen L , Morren MA . Topical corticosteroid phobia among healthcare professionals using the TOPICOP score. Acta Derm Venereol. 2019;99(11):1004–8. 10.2340/00015555-3220 31099401

[10] Farrugia LL , Lee A , Fischer G , Blaszczynski A , Carter SR , Smith SD , et al. Evaluation of the influence of pharmacists and GPs on patient perceptions of long‐term topical corticosteroid use. J Dermatol Treat. 2017;28(2):112–8. 10.1080/09546634.2016.1213353 27424888

[11] Smith SD , Harris V , Lee A , Blaszczynski A , Fischer G . General practitioners knowledge about use of topical corticosteroids in paediatric atopic dermatitis in Australia. Aust Fam Physician. 2017;46(5):335–40. 28472581

[12] Charman CR , Morris AD , Williams HC . Topical corticosteroid phobia in patients with atopic eczema. Br J Dermatol. 2000;142(5):931–6. 10.1046/j.1365-2133.2000.03473.x 10809850

[13] Verberne LVR , Flinterman LE . Huisartsenzorg voor patiënten met eczeem. Utrecht: Nivel; 2020.

[14] Kamphuis ECM , Deurloo J , Dirven‐Meijer P , van Gameren H , Nawijn L , van Hees C , et al. Huidafwijkingen. Utrecht: Nederlands Centrum Jeugdgezondheid; 2012.

[15] Siderius EJ , Carmiggelt B , Rijn CS , Heerkens YF . Preventive Child Health Care within the framework of the Dutch Health Care System. J Pediatr. 2016;177S:S138–S41. 10.1016/j.jpeds.2016.04.050 27666262

[16] Thormann K , Aubert H , Barbarot S , Britsch‐Yilmaz A , Chernyshov P , Deleuran M , et al. Position statement on the role of nurses in therapeutic patient education in atopic dermatitis. J Eur Acad Dermatol Venereol. 2021;35(11):2143–8. 10.1111/jdv.17487 34289187

[17] Smith SD , Lee A , Blaszczynski A , Fischer G . Pharmacists' knowledge about use of topical corticosteroids in atopic dermatitis: pre and post continuing professional development education. Australas J Dermatol. 2016;57(3):199–204. 10.1111/ajd.12339 25846602

[18] Brusamento S , Kyaw BM , Whiting P , Li L , Tudor Car L . Digital health professions education in the field of pediatrics: systematic review and meta‐analysis by the digital health education collaboration. J Med Internet Res. 2019;21(9):e14231. 10.2196/14231 31573906 PMC6785725

[19] Oppong R , Smith RD , Little P , Verheij T , Butler CC , Goossens H , et al. Cost‐effectiveness of internet‐based training for primary care clinicians on antibiotic prescribing for acute respiratory tract infections in Europe. J Antimicrob Chemother. 2018;73(11):3189–98. 10.1093/jac/dky309 30165522

[20] Kinderdermatologie . Masterclass voor zorgprofessionals in de JGZ. Captise; 2020. https://www.captise.nl/Zorg‐Jeugd/ArtMID/504/ArticleID/4463/Kinderdermatologie‐Masterclass‐voor‐zorgprofessionals‐in‐de‐JGZ

[21] Dirven‐Meijer PCDKC , Nonneman MMG , Van Sleeuwen D , De Witt‐de Jong AWF Burgers JSOW , De Vries CJH . NHG‐Standaard Eczeem. Huisarts Wet; 2014. p. 240–52.

[22] NVDV . Richtlijn Constitutioneel eczeem; 2019.

[23] NVDV . Leidraad Dermatocorticosteroïden NVDV; 2019.

[24] Bewley A , Dermatology Working Group . Expert consensus: time for a change in the way we advise our patients to use topical corticosteroids. Br J Dermatol. 2008;158(5):917–20. 10.1111/j.1365-2133.2008.08479.x 18294314

[25] Moret L , Anthoine E , Aubert‐Wastiaux H , Le Rhun A , Leux C , Mazereeuw‐Hautier J , et al. TOPICOP©: a new scale evaluating topical corticosteroid phobia among atopic dermatitis outpatients and their parents. PLoS One. 2013;8(10):e76493. 10.1371/journal.pone.0076493 24146878 PMC3797828

[26] Cohen J . Statistical power analysis for the behavioral sciences. 2nd ed ed. New York: Academic Press.; 1988.

[27] Tudor Car L , Soong A , Kyaw BM , Chua KL , Low‐Beer N , Majeed A , et al. Health professions digital education on clinical practice guidelines: a systematic review by Digital Health Education collaboration. BMC Med. 2019;17(1):139. 10.1186/s12916-019-1370-1 31315642 PMC6637541

[28] Koster ES , Philbert D , Zheng X , Moradi N , de Vries TW , Bouvy ML , et al. Reducing corticosteroid phobia in pharmacy staff and parents of children with atopic dermatitis. Int J Clin Pharm. 2021;43(5):1237–44. 10.1007/s11096-021-01241-2 33582952 PMC8460576

[29] Lugtenberg M , Zegers‐van Schaick JM , Westert GP , Burgers JS . Why don't physicians adhere to guideline recommendations in practice? An analysis of barriers among Dutch general practitioners. Implement Sci. 2009;4(1):54. 10.1186/1748-5908-4-54 19674440 PMC2734568

[30] Lugtenberg M , Burgers JS , Han D , Westert GP . General practitioners' preferences for interventions to improve guideline adherence. J Eval Clin Pract. 2014;20(6):820–6. 10.1111/jep.12209 24953439

[31] Institute of Medicine . Chapter 6 promotion of adoption of clinical practice guidelines. In: Graham R , Mancher M , Wolman DM , Greenfield S , Steinberg E , editors. Clinical practice guidelines we can trust. Washington, DC: The National Academies Press; 2011. p. 145. 24983061

[32] Puar N , Chovatiya R , Paller AS . New treatments in atopic dermatitis. Ann Allergy Asthma Immunol. 2021;126(1):21–31. 10.1016/j.anai.2020.08.016 32818591

[33] Veenje S , Osinga H , Antonescu I , Bos B , de Vries TW . Focus group parental opinions regarding treatment with topical corticosteroids on children with atopic dermatitis. Allergol Immunopathol. 2019;47(2):166–71. 10.1016/j.aller.2018.05.007 30316560

