# Supplementary material for: Long term effects of digital education among healthcare professionals in paediatric dermatology: Opportunities for improving care
Source: Skin Health Dis. 2022 Jul 20;2(3):e143. doi: 10.1002/ski2.143 (PMC9435444; doi:10.1002/ski2.143)
Supplement: Supplementary file 1 — Supporting Information S1 [file SKI2-2-e143-s001.docx]

## **Supporting information**

Appendix S1. Role of ‘youth health care physicians’ in the Netherlands.

In the Netherlands each municipality has a dedicated youth health team set out to monitor the health, growth and development of children up to the age of 16. Between birth and the age of four children are invited to attend 13 check-ups. During these visits, children undergo sight and hearing tests, growth monitoring (health and weight), and speech and motor development checks. These check-ups are supervised by youth health care physicians. When children show any signs of a possible disease youth health care physicians will perform a thorough examination. Based on the findings youth health care physicians can take several actions:

1. Suggest caregivers to contact the general practitioner.
2. Inform and refer to the general practitioner.
3. Inform and refer directly to a medical specialist, i.e. a pediatrician, dermatologist.
4. Provide education, i.e. on the use of emollients in atopic dermatitis.

Additionally in some municipalities the youth health care physicians prescribe treatment. However, more importantly, youth health care physicians are trusted by caregivers and are very accessible for questions. Caregivers attending these check-ups frequently ask youth health care physicians for medical advice and their vision on the treatment of their child.

With up to 20% of children having atopic dermatitis and reluctance for topical corticosteroids among caregivers, youth health care physicians play an important role in enhancing adherence and thereby outcomes.

### Appendix S2. Questionnaire (translated from Dutch to English)

**Part I**

1. What is your age?

……….. years.

1. What is your gender? (Check one box)

- Female
- Male

1. What is your profession? (Check one box)

- Pediatrician
- Resident in pediatrics
- Youth health care physicians care
- Resident in youth health care
- Nurse in youth health care
- Nurse in dermatology
- Physician in dermatology
- Nurse in pediatrics
- Other

1. How often do you prescribe topical corticosteroids or do you give information about the use of topical corticosteroids to patients? (Check one box)

- (Almost) never
- Monthly
- Weekly
- Daily

1. Which source of information do you use to get prescription information about topical corticosteroids/ do you base your counselling on? (More answers applicable)

- Medical guidelines (NHG, NVK, etc.)
- Scientific journals
- Own experience
- Clinical meetings, congresses or courses
- Information pamphlets
- Commercial material of pharmaceutical industries

1. What duration do you generally advise to use topical corticosteroids for a 3 year old child with moderate-severe atopic dermatitis? (Check one box)

- Until patches are gone (in a tapering scheme for example)
- Maximum duration: 2 weeks
- Maximum duration: 1 month
- Other…

1. Which application instructions do you give during your counseling about topical corticosteroids? (Check one box)

- I don’t give information on the use of topical corticosteroids
- Apply in a thin layer
- Apply according to the Finger Tip Unit
- Apply in a thick layer

1. What duration of a tube with topical corticosteroids (30 grams) would you advise for a ten year old child with atopic dermatitis on the arms, elbow pits and knee pits (6% of the total body surface) if the child were to apply it once a day? (Check one box)

- 1 week
- 2 weeks
- 1 month
- Longer than 1 month

1. Are you familiar with the Finger Tip Unit (FTU)? (Check one box)

- Yes
- No

1. Are you familiar with the different classes of potency in topical corticosteroids used in the Netherlands? (Check one box)

- Yes
- No

1. Are you familiar with the concept of a tapering scheme in the treatment of atopic dermatitis with topical corticosteroids?

- Yes
- No

### **Part II**

### **TOPICOP-P**

For every statement, you can assume the topical corticosteroids (TCS) are used according to the guidelines (for example, using mometasone ointment (class III) in the treatment of a child with moderate-severe atopic dermatitis). Please select one box per statement

1. TCS pass into the blood stream and cause systemic effects.

- Totally disagree
- Disagree
- Agree
- Totally agree

1. TCS can lead to (skin)infections.

- Totally disagree
- Disagree
- Agree
- Totally agree

1. TCS make you fat.

- Totally disagree
- Disagree
- Agree
- Totally agree

1. TCS damage the skin.

- Totally disagree
- Disagree
- Agree
- Totally agree

1. TCS will affect the future health.

- Totally disagree
- Disagree
- Agree
- Totally agree

1. TCS can lead to asthma.

- Totally disagree
- Disagree
- Agree
- Totally agree

1. Even though I don't know all the side effects, I'm reluctant to use TCS myself or allow patients to use it..

- Totally disagree
- Disagree
- Agree
- Totally agree

1. I discourage applying too much TCS.

- Never
- Sometimes
- Often
- Always

1. I discourage using TCS on certain zones like the eyelids.

- Never
- Sometimes
- Often
- Always

1. I recommend waiting as long as possible before starting TCS.

- Never
- Sometimes
- Often
- Always

1. I recommend stopping TCS as soon as possible.

- Never
- Sometimes
- Often
- Always

1. I provide additional reassurance and confirmation regarding the use of TCS.

- Never
- Sometimes
- Often
- Always

**Part III – (6 months after education)**

This is the last part of this questionnaire. Following are some statements about topical corticosteroids.

1. Has the masterclass changed your prescription practices or patient education about topical corticosteroids? Please explain (open question)

……………………………………………………………………………………….

1. Since the masterclass, I have been providing more information about topical corticosteroids.
   - Strongly disagree
   - Disagree
   - Neither agree nor disagree
   - Agree
   - Strongly agree
2. Since the master class, I refer patients less often (to a dermatologist).
   - Strongly disagree
   - Disagree
   - Neither agree nor disagree
   - Agree
   - Strongly agree
3. Please select statements that applied to you if your practices have changed since the masterclass (multiple answers possible)
   - I more often prescribe potent topical corticosteroids
   - I recommend applying more topical corticosteroids (smearing thicker)
   - I make more use of tapering schemes
   - I make more use of preventive treatment (pulse-therapy – preventive treatment with the application of a mild to moderate potent topical corticosteroid)
   - I am more skilled in finding potential pitfalls in the treatment of patients
   - I feel more competent in treating patients

Table S3. Effects of digital education on prescription practices and patient education.

| Item | Pre-education  (N=66) | Post-education  (N=76) | Six months after education  (N=34) | Pre vs.  Post^a^ | | Pre vs.  Follow-up^b^ | | Post vs.  Follow-up^c^ | |
| --- | --- | --- | --- | --- | --- | --- | --- | --- | --- |
|  | n (%) | n (%) | n (%) | Effect size (φc) | P | Effect size (φc) | P | Effect size (φc) | P |
| **Familiarity with FTU (overall)** | 43 (69) | 76 (100) | 33 (100) | **0.44** | **<.01** | **0.37** | **<.01** | - | - |
| Pediatricians | 17 (89) | 25 (100) | 6 (100) |  |  |  |  |  |  |
| Youth health care physicians | 17 (57) | 35 (100) | 19 (100) |  |  |  |  |  |  |
| Nurses^d^ | 9 (69) | 16 (100) | 8 (100) |  |  |  |  |  |  |
| **Familiarity with classes of potency** | 48 (76) | 75 (99) | 31 (94) | **0.35** | **<.01** | **0.22** | **.05** | 0.13 | .22 |
| Pediatricians | 18 (95) | 25 (100) | 6 (100) |  |  |  |  |  |  |
| Youth health care physicians | 23 (77) | 35 (100) | 17 (89) |  |  |  |  |  |  |
| Nurses^d^ | 7 (50) | 15 (94) | 8 (100) |  |  |  |  |  |  |
| **Familiarity with tapering schemes** | 56 (88) | 75 (99) | 32 (97) | **0.23** | **.01** | **0.16** | **.16** | 0.06 | .50 |
| Pediatricians | 18 (95) | 25 (100) | 6 (100) |  |  |  |  |  |  |
| Youth health care physicians | 27 (87) | 34 (97) | 18 (95) |  |  |  |  |  |  |
| Nurses^d^ | 11 (79) | 16 (100) | 8 (100) |  |  |  |  |  |  |
| **Duration of a tube TCS**:  1-2 weeks*†*  1 month or longer | 27 (45)  33 (55) | 69 (91)  7 (9) | 29 (88)  4 (12) | **0.50** | **<.01** | **0.42** | **<.01** | 0.04 | .73 |
| Pediatricians (correct) | 11 (58) | 23 (92) | 5 (83) |  |  |  |  |  |  |
| Youth health care physicians (correct) | 12 (40) | 32 (91) | 16 (84) |  |  |  |  |  |  |
| Nurses^d^ (correct) | 4 (29) | 14 (88) | 8 (100) |  |  |  |  |  |  |
| **Treatment duration of TCS**:  Until patches are gone*†*  Maximum duration 2 -4 weeks | 50 (93)  2 (7) | 73 (100)  0 (0) | 33 (100)  0 (0) | **0.21** | **.03** | 0.16 | .29 | - | - |
| Pediatricians (correct) | 18 (95) | 25 (100) | 6 (100) |  |  |  |  |  |  |
| Youth health care physicians (correct) | 22 (92) | 33 (100) | 15 (100) |  |  |  |  |  |  |
| Nurses^d^ (correct) | 10 (91) | 15 (100) | 7 (100) |  |  |  |  |  |  |
| **Application instructions**:  No instructions  Thin  According FTU†  Thick | 15 (24)  13 (21)  35 (56)  0 (0) | 1 (1)  0 (0)  75 (99)  0 (0) | 2 (7)  0 (0)  31 (94)  0 (0) | **0.53** | **<.01** | **0.40** | **<.01** | 0.13 | .22 |
| Pediatricians (correct) | 16 (84) | 25 (100) | 6 (100) |  |  |  |  |  |  |
| Youth health care physicians (correct) | 11 (58) | 34 (100) | 17 (100) |  |  |  |  |  |  |
| Nurses^d^ (correct) | 8 (73) | 16 (100) | 8 (100) |  |  |  |  |  |  |

Percentages and numbers may not add up because of missing values a) Responses before digital education compared to responses directly after digital education. b) Responses before digital education compared to responses 6 months after education c) Responses directly after digital education compared to responses 6 months after education† Correct response. d) nurses consisted out of nurses and nurse practitioners (2 pre and post-education, 1 after 6 months of follow-up

| Item | Six months after education (N=34) |
| --- | --- |
|  | n (%) |
| 1. Self-perceived change in prescription practices or patient education | 13 (68) |
| 1. Provision of more patient education on the use of TCS | 12 (39) |
| 1. Less referrals | 3 (10) |
|  | |
| - I feel more competent in treating patients | 8 (89) |
| - I more often prescribe potent topical corticosteroids | 2 (22) |
| - I recommend applying more topical corticosteroids (smearing thicker) | 3 (33) |
| - I make more use of tapering schemes | 3 (33) |
| - I make more use of preventive treatment (pulse-therapy – preventive treatment with the application of a mild to moderate potent topical corticosteroid) | 2 (22) |
| - I am more skilled in finding potential pitfalls in the treatment of patients | 4 (44) |

Table S4. Perceived effects of education after six months

Percentages and numbers may not add up because of missing values
